# Electric Field‐Induced Nonreciprocal Directional Dichroism in a Time‐Reversal‐Odd Antiferromagnet

**DOI:** 10.1002/adma.202414876

**Published:** 2025-01-15

**Authors:** Takeshi Hayashida, Koei Matsumoto, Tsuyoshi Kimura

**Affiliations:** ^1^ Department of Applied Physics University of Tokyo Bunkyo‐ku Tokyo 113‐8656 Japan

**Keywords:** altermagnet, antiferromagnet, domain, nonreciprocal optical phenomena

## Abstract

Antiferromagnets with broken time‐reversal (T) symmetry (T‐odd antiferromagnets) have gained extensive attention, mainly due to their ferromagnet‐like behavior despite the absence of net magnetization. However, certain types of T‐odd antiferromagnets remain inaccessible by the typical ferromagnet‐like phenomena (e.g., anomalous Hall effect). One such system is characterized by a T‐odd scalar quantity, the magnetic toroidal monopole. To access the broken T symmetry in such a system, a unique nonreciprocal optical phenomenon, electric field‐induced nonreciprocal directional dichroism (*E*‐induced NDD), is employed. Signals of *E*‐induced NDD are successfully detected in a T‐odd antiferromagnet, Co₂SiO₄, whose magnetic structure is characterized by the magnetic toroidal monopole. Furthermore, by spatially resolving the *E*‐induced NDD, spatial distributions of a pair of domain states related to one another by the T operation are visualized. The domain imaging revealed the inversion of the domain pattern by applying a magnetic field, which is explained by trilinear coupling attributed to the piezomagnetic effect. The observation of *E*‐induced NDD highlights unique functionalities of T‐odd antiferromagnets.

## Graphical abstract

Electric field‐induced nonreciprocal directional dichroism (*E*‐induced NDD) ascribed to the diagonal electrotoroidic effect is discovered in a time‐reversal‐odd antiferromagnet Co₂SiO₄ possessing a time‐reversal‐odd scalar quantity, magnetic toroidal monopole. Spatial distribution measurements of *E*‐induced NDD reveal domain structures. Magnetic field‐induced domain inversion mediated by the piezomagnetic effect highlights the rich physics of time‐reversal‐odd antiferromagnets.

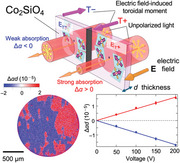

## 1 Introduction

Antiferromagnetism is a state in which magnetic moments are ordered in a way that the overall magnetization is canceled out. Although Louis Néel,^[^ ^1^ ^]^ who was awarded the Nobel Prize for research on antiferromagnetism in 1970, described it as “interesting but useless,” a considerable amount of research since his comment has revealed that certain antiferromagnets exhibit unique functionalities or physical phenomena. These properties often arise from the breaking of time‐reversal (T) symmetry. For example, in an insulating antiferromagnet where space‐inversion (P) and T symmetries are simultaneously broken but the combined PT symmetry is preserved, the linear magnetoelectric (ME) effect emerges.^[^ ^2^, ^3^, ^4^ ^]^ Such ME antiferromagnets also exhibit unique nonreciprocal optical and transport properties.^[^ ^5^ ^]^ For another example, recent investigations have revealed that antiferromagnets breaking T and PT symmetries show physical properties that are usually observed in ferromagnets, including the anomalous Hall effect, spontaneous magneto‐optical Kerr effect, and nonrelativistic spin splitting, despite the absence of (or tiny) net magnetization.^[^ ^6^, ^7^, ^8^, ^9^, ^10^, ^11^, ^12^, ^13^, ^14^, ^15^ ^]^ To understand such unconventional properties in T‐ and PT‐odd antiferromagnets with collinear and compensated magnetism, a novel concept termed “altermagnetism” has been introduced.^[^ ^16^, ^17^ ^]^ Notably, altermagnetism has attracted extensive interest partly because of the above‐mentioned ferromagnet‐like properties.

Herein, we examine another unique property of T‐ and PT‐odd antiferromagnets, electric field‐induced (*E*‐induced) nonreciprocal directional dichroism (NDD), where the application of *E* induces asymmetry in optical absorption between two counterpropagating light beams. Using this unique nonreciprocal optical phenomenon, we successfully detect the order parameter of a T‐ and PT‐odd antiferromagnet and access its domain states.

## 2 Electrotoroidic Effect and Electric Field‐Induced Nonreciprocal Directional Dichroism

### 2.1 Electrotoroidic Effect

**Figure 1:**
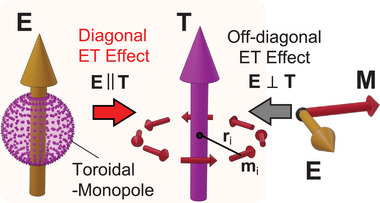
Magnetic toroidal moment and electrotoroidic (ET) effect. Magnetic toroidal moment **T** is composed of vortex arrangements of magnetic dipoles **m** *_i_* (middle panel). **T** can be induced by an electric field **E** as a result of the ET effect. The off‐diagonal ET effect refers to **T** induced perpendicular to **E** (**E**⊥**T**), which is achieved by a cross‐product of magnetization **M** and an applied **E**, **T**∝**M**×**E** (right panel). The diagonal ET effect, **T** induced parallel to **E** (**E**||**T**), is mediated by a T‐odd scalar quantity, such as magnetic toroidal monopole (left panel).

The targeted class of materials in this research is an antiferromagnet that breaks T symmetry, but the above‐mentioned ferromagnet‐like behaviors are symmetrically forbidden. Even in such materials, however, T‐odd property still hosts interesting physical phenomena. One such phenomenon is the diagonal electrotoroidic (ET) effect,^[^ ^18^, ^19^ ^]^ where the application of an electric field **E** induces magnetic toroidal moment **T** in the direction parallel to **E**. **T** is a T‐odd polar vector that describes vortex arrangements of magnetic dipoles as $T\propto \sum_{i}r_{i}\times m_{i}$, where **r** *_i_* is the position vector of the magnetic dipole **m** *_i_* at the *i* site with reference to the high symmetry point (middle panel of **Figure** **1**). The spontaneous ferroic order of magnetic toroidal moments, so‐called ferrotoroidic order, has long been discussed theoretically,^[^ ^18^, ^20^, ^21^, ^22^ ^]^ and ferrotoroidic domains were experimentally observed using optical second harmonic generation in 2007.^[^ ^23^ ^]^ In parallel with the discussion on the ferrotoroidic order, Schmid introduced a linear response of **T** to applied **E**, described as *T_i_* = *θ_ij_E_j_* and termed it the ET effect.^[^ ^18^, ^19^ ^]^ Here, *θ_ij_* is a second rank T‐odd polar tensor called the ET tensor. Note that the ET effect is a phenomenon ascribed to a bi‐linear ME coupling since **T** is symmetrically equivalent to **H**×**E**, where **H** is a magnetic field, and the coupling between **T** and **E** is expressed as (**H**×**E**)**·E**.^[^ ^18^, ^24^ ^]^ In Note S1 (Supporting Information) of Supporting Information, we summarize the phenomenology of the ET effect based on free energy. When *i* ≠ *j* and *i *= *j*, the effect is off‐diagonal (**E**⊥**T**) and diagonal (**E**||**T**), respectively. Off‐diagonal coupling is rather common and is observed in systems breaking both P and T symmetries with the cross product of spontaneous (or field‐induced) **M** and an applied **E**, i.e., **T** ∝ **M**×**E** (right panel of Figure 1). The off‐diagonal ET effect has been observed through nonreciprocal optical phenomena in systems with **E** and **H** applied in perpendicular directions (**T** ∝ **H**×**E**).^[^ ^25^ ^]^

On the other hand, for the emergence of the diagonal ET effect, **T** is induced as a result of changing only the T symmetry (T‐even to T‐odd) while maintaining the direction of **E**, which should be observed in systems holding a T‐odd “scalar” quantity. Recently, Hayami and Kusunose showed that such a class is characterized by “magnetic toroidal monopole”, that is, all‐in or all‐out arrangements of the local magnetic toroidal moment^[^ ^26^ ^]^ (left panel of Figure 1). Considering symmetry, 32 magnetic point groups in yellow‐colored rows of Table S1 (Supporting Information) allow for the emergence of the magnetic toroidal monopole and therefore the diagonal ET effect.^[^ ^26^ ^]^ (In this paper, the term “magnetic point groups” is used to refer to all 122 Shubnikov groups including 32 crystallographic point groups (Shubnikov type I), 32 gray groups (type II), and 58 black‐and‐white groups (type III).) More recently, a similar classification focusing on spontaneous and field‐induced **T** has been introduced by Xu and coworkers.^[^ ^27^ ^]^ We note that the systems described by the magnetic toroidal monopole can also be described by the magnetic octupole.^[^ ^28^, ^29^ ^]^ The magnetic toroidal monopole better reflects T‐odd scalar quantity that we focus on in this research, and thus we adopt the classification using the magnetic toroidal monopole. We also note that the bi‐linear ME coupling is suggested as an effective but unexplored way to approach magnetic octupole orders.^[^ ^30^ ^]^

### 2.2 Electric Field‐Induced Nonreciprocal Directional Dichroism

In contrast with the measurement of **M**, that of **T** is non‐trivial, and thus methods to observe the diagonal ET effect have been less established. However, in systems with finite **T**, the characteristic optical phenomenon, NDD, emerges. NDD has been observed in ferrotoroidic materials and materials in which magnetic toroidal moment is induced by the cross product of **E** (**P**) and **H** (**M**).^[^ ^31^, ^32^, ^33^, ^34^ ^]^ Because the states with opposite signs of **T**, T+, and T−, show different absorption, NDD has been used to visualize ferrotoroidic domains.^[^ ^35^, ^36^ ^]^ In the system where the diagonal ET effect, i.e., *E*‐induced **T**, is allowed, therefore, the emergence of *E*‐induced NDD is expected.

*E*‐induced NDD will be described as the changes in an absorption coefficient *α* (cm^−1^) under **E**, and expressed as

(1) $$\begin{array}{c} \alpha =\alpha_{0}+\Delta \alpha =\alpha_{0}+\beta \left(k\cdot E\right) \end{array}$$

where *α*_0_ is an absorption coefficient without **E**, Δ*α* is the difference in the absorption coefficient induced by **E**, *β* is the coefficient describing the *E*‐induced NDD, and **k** is the light propagation vector. For the derivation of Equation (1) based on the optical ME coupling, see Note S2 (Supporting Information). When **E** is parallel to **k**, the intensity of the transmitted light *I* is described as:

(2) $$\begin{array}{c} I=I_{0}\;e^{-\left(\alpha_{0}+\Delta \alpha \right)d}\approx I_{0}e^{-\alpha_{0}d}\left(1-\Delta \alpha d\right)\\ =I_{0}e^{-\alpha_{0}d}\left(1-\beta V\right) \end{array}$$

where *I*_0_ is the intensity of the incident light, *d* (cm) is the sample thickness, and *V *= *Ed* (V) is the applied voltage. In the transformation of Equation (2), Δ*αd* is assumed to be small. Under this approximation, the change in transmitted light intensity is linear relative to *V*. Note that this *E*‐induced NDD is understood as the coupling of (**H**_ω_×**E**_ω_)**·E**, where **E**_ω_ and **H**_ω_ are oscillating electric field and magnetic field of light, respectively. Thus, *E*‐induced NDD is also a type of bi‐linear (optical) ME coupling.

### 2.3 Time‐Reversal‐Symmetry‐Broken Antiferromagnet Co_2_SiO_4_

**Figure 2:**
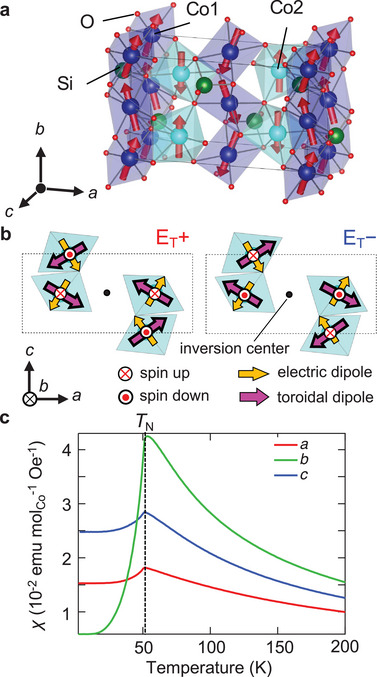
a) Crystal and magnetic structures of Co_2_SiO_4_. The red arrows denote Co^2+^ spins. b) Two magnetic domain states (E_T_+ and E_T_−) related to one another via time‐reversal operation. For clarity, only CoO_6_ octahedral units at the Co2 sites are shown. The circles with red dots or crosses in the center, orange arrows, and magenta arrows denote local spins, electric dipoles, and magnetic toroidal dipoles, respectively. The E_T_+ and E_T_− domain states are characterized by magnetic toroidal monopoles with a positive ($\sum_{i}r_{i}\cdot t_{i}>0$) and negative ($\sum_{i}r_{i}\cdot t_{i}<0$) divergence of **t**, respectively. The dashed box denotes a unit cell. c) Temperature profiles of magnetic susceptibility *χ* along the *a*, *b*, and *c* axes.

To manifest the *E*‐induced NDD, we selected Co_2_SiO_4_ as the target material. Co_2_SiO_4_ crystalizes in the olivine structure (space group *Pnma*, **Figure** **2**a).^[^ ^37^, ^38^, ^39^ ^]^ Co^2+^ ions are surrounded by six O^2−^ ions and occupy two different octahedral sites: Co1 located at the centrosymmetric site (site symmetry $\bar{1}$) and Co2 located at the mirror‐symmetric site whose mirror plane is perpendicular to the *b* axis (site symmetry *m*). Co_2_SiO_4_ shows an antiferromagnetic (AFM) transition at *T*_N_ ≈50 K^[^ ^40^, ^41^, ^42^ ^]^ where the magnetic point group changes from T‐even *mmm*1′ (Shubnikov type II) to T‐odd *mmm* (Shubnikov type I). Figure 2c shows the temperature profile of magnetic susceptibility of our Co_2_SiO_4_ crystal. In the AFM phase, spins on the Co2 sites are collinearly aligned along the *b*‐axis, whereas those on the Co1 sites are slightly tilted from the *b*‐axis and form zigzag spin chains (red arrows in Figure 2a). The magnetic point group *mmm* in the AFM phase holds the magnetic toroidal monopole^[^ ^26^ ^]^ and permits the diagonal ET effect with the nonzero distinct ET tensor components of *θ*_11_, *θ*_22_, and *θ*_33_^[^ ^43^ ^]^ (Note S3, Supporting Information). Although the noncollinear spins on the Co1 sites are also related to the breaking of T symmetry and the diagonal ET effect,^[^ ^27^ ^]^ herein, we focused on the collinear spins on the Co2 site for simplification. As mentioned above, **T** describes vortex arrangements of multiple magnetic dipoles; however, local magnetic toroidal moment **t** can also be defined at a single ion site by considering the cross product of a local electric dipole moment **p** and a local magnetic moment **m**, as **t** ∝ **p**×**m**.^[^ ^34^ ^]^ At the Co2 site with **m**_Co2_ (red arrows in Figure 2b), an inversion symmetry is locally broken while a mirror symmetry perpendicular to the *b* axis is preserved, and thus **p**_Co2_ emerges in the *ac* plane (orange arrows in Figure 2b). Then, a local magnetic toroidal moment at the Co2 site, **t**_Co2_ (∝ **p**_Co2_×**m**_Co2_) is introduced (magenta arrows in Figure 2b). Note that the sum of **t**_Co2_ is canceled out in a unit cell with four Co2 sites; therefore, Co_2_SiO_4_ is not ferrotoroidic (***T* **= **0**) in the absence of **E**. We also note that spin‐up and spin‐down sublattices are related to each other by glide operations but not by space‐inversion or translation, which satisfies the requirement of altermagnetism from the viewpoint of symmetry,^[^ ^44^, ^45^ ^]^ i.e., Co_2_SiO_4_ is an altermagnet candidate. Magnetic point groups of representative candidates for altermagnets such as RuO_2_ (4′/*mmm*′), Mn_5_Si_3_ ($\bar{1}$), and MnTe (*m*′*m*′*m*) permit either or both diagonal and off‐diagonal ET effects, depending on their symmetry (Table S1, Supporting Information).

**Figure 3:**
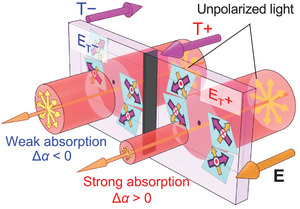
*E*‐induced NDD in Co_2_SiO_4_. Under an electric field **E**, the E_T_+ and E_T_− domain states exhibit magnetic toroidal moments with opposite polarities (T+ and T−), resulting in an absorption difference of unpolarized light. Although here we depict the model in the setting of **E**||**k**||*b*, *E‐*induced NDD is permitted in any direction if **E**||**k** is satisfied.

In the absence of **E**, thus, the AFM phase of Co_2_SiO_4_ does not show finite **T** but holds a T‐odd scalar quantity, that is, magnetic toroidal monopole ($\propto \sum_{i}r_{i}\cdot t_{i}$).^[^ ^26^ ^]^ One can consider the two domain states related to each other by the T operation, termed E_T_+ and E_T_– domains (left and right panels of Figure 2b), which are characterized by the magnetic toroidal monopole with positive ($\sum_{i}r_{i}\cdot t_{i}>0$) and negative ($\sum_{i}r_{i}\cdot t_{i}<0$) divergence, respectively. The sign of the ET tensors of the two E_T_ domain states is opposite, indicating that these states exhibit **T** with opposite polarities (T+ and T−) under **E**. This results in the opposite sign of *β* in Equation (2), i.e., the two domain states show different absorption under **E**. **Figure** **3** schematically illustrates the ET effect and the *E*‐induced NDD expected in a multidomain state of Co_2_SiO_4_. The way to align the system into a single domain state is nontrivial. Therefore, spatially resolved measurements are suitable for the detection of *E*‐induced NDD.

## 3 Results and Discussion

### 3.1 Observation of *E*‐Induced NDD

**Figure 4:**
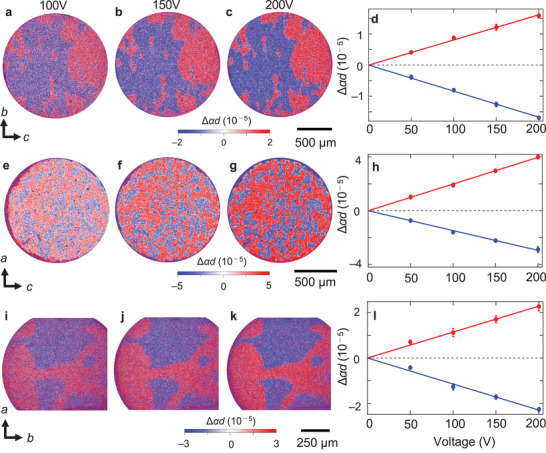
*E*_T_ domains observed by *E*‐induced NDD. a–c), e–g), and i–k) show maps of changes in the absorption of unpolarized light (Δ*αd*) induced by applying voltage in the (100), (010), and (001) planes, respectively. The first (a,e,i), second (b,f,j), and third (c,g,k) columns show the images obtained under applied voltages of 100, 150, and 200 V. respectively. The applied voltage and light propagation direction **k** were parallel to the normal axis of each plane. The wavelengths of the incident light were 550 nm [(a)‐(c) and (i)‐(k)] and 590 nm [(e)‐(g)]. The images were obtained at 4 K after cooling the crystals at zero magnetic field. d), h), and (l) show the voltage dependence of the Δ*αd* averaged in 10 different points for each domain state in the (100), (010), and (001) planes, respectively. The sampling positions are shown in Figure S1 of Note S4 (Supporting Information). The red and blue dots correspond to the data for the positive and negative Δ*αd* single domain areas, respectively. The error bars show the standard deviations for the 10 points. The lines denote the least‐squares fitting lines.

**Figure 5:**
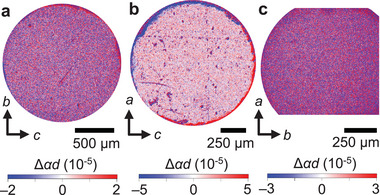
Spatial maps of Δ*αd* above *T*_N_. a), b), and (c) show the maps of changes in Δ*αd* induced by an applied voltage of *V* = 200 V in the (100), (010), and (001) planes, respectively, at temperatures above *T*_N_ = 50 K ((a):52 K, (b):61 K, (c):53 K). Applied electric field **E** and light propagation direction **k** are parallel to the normal axis of each plane. The wavelengths of the incident light are 550 nm (a) and (c) and 590 nm (b). The images in panels (a), (b), and (c) were taken at the same position on the same sample as those in Figure 4a–c, e–g, and i–k, respectively.

We measured *E*‐induced NDD of Co_2_SiO_4_ single crystals. For the measurements in the transmittance geometry, we prepared three thin samples whose widest faces are parallel to the (100), (010), and (001) planes (see Section 5, Experimental Section). Based on other *E*‐induced optical effects in magnetic materials (e.g., *E*‐induced magnetic circular dichroism in Cr_2_O_3_^[^ ^46^ ^]^), *E*‐induced NDD signals are expected to be weak. Thus, we adopted an electric field‐modulation imaging technique,^[^ ^47^ ^]^ which enables us to detect the spatial distribution of *E*‐induced NDD signals with high sensitivity (see Section 5, Experimental Section). **Figure** **4**a–c, e–g, i–k show the maps of Δ*αd* obtained in the geometry where both **k** and **E** are along the *a*, *b*, and *c* axes, respectively, at selected *V*. The images were obtained at 4 K (< *T*_N_) using unpolarized light with wavelengths of 550 nm (Figure 4a–c, i–k) and 590 nm (Figure 4e–g). In all the samples, a clear contrast of red and blue corresponding to the positive and negative Δ*α*, respectively, is observed at 200 V (Figure 4c,g,k). The color contrasts monotonically increase with voltage in all the geometries. We calculated the average Δ*αd* for the pixels at selected single domain areas (both positive and negative Δ*αd*) denoted by boxes in Figure S1a–c (Supporting Information) and determined the *V* dependence of Δ*αd*. As shown in Figure 4d,h,l, Δ*αd* is linear with respect to *V*. We also checked that the contrasts disappeared at temperatures above *T*_N_ (**Figure** **5**). Furthermore, the lock‐in measurements with a focused laser beam in a detailed temperature range confirm that the *E*‐induced NDD disappears above *T*_N_ (Figure S2 in Note S5, Supporting Information). Consequently, the obtained contrasts are pairs of E_T_ domains showing the opposite sign of *E*‐induced NDD resulting from T symmetry breaking. The effect is observed in all the settings of **E**||**k**||*a*, *b*, *c*, which is consistent with the magnetic point group *mmm* where all the diagonal ET tensor components (*θ*_11_, *θ*_22_, and *θ*_33_) can be finite. Note that the signs of the domain states between the different planes cannot be uniquely associated in the present measurements; thus, the color (the sign of Δ*α*) coincidence of the domains between Figure 4c,g,k does not necessarily indicate the coincidence of the domain states.

The domain sizes on the (100) and (001) planes (Figure 4c,k) are on the order of several hundred micrometers, whereas that on the (010) plane (Figure 4g) are on the order of several tens of micrometers. The domains on the (010) plane are expected to be vertically cut from the domains on the (100) and (001) faces along the *b*‐axis, which does not match the experimental results. As discussed below, the domains of Co_2_SiO_4_ are probably related to strain, and the domain size may be affected by residual strains induced during sample preparation, particularly, cutting and polishing.

We have also acquired the spectra of *E*‐induced NDD by focusing a laser beam on a single domain region and using a lock‐in technique (Figure S3, Supporting Information). Although NDD itself does not depend on the polarization of incident light, polarization affects the absorption and *E*‐induced NDD because of the orthorhombic crystal structure. Therefore, the measurements were performed using linearly polarized light. Complex peak structures of *E*‐induced NDD were observed in the energy range of the Co *d*–*d* transition.^[^ ^48^ ^]^ For a detailed analysis and discussion of the spectra, see Note S6 (Supporting Information).

### 3.2 Domain Inversion by Applying a Magnetic Field

**Figure 6:**
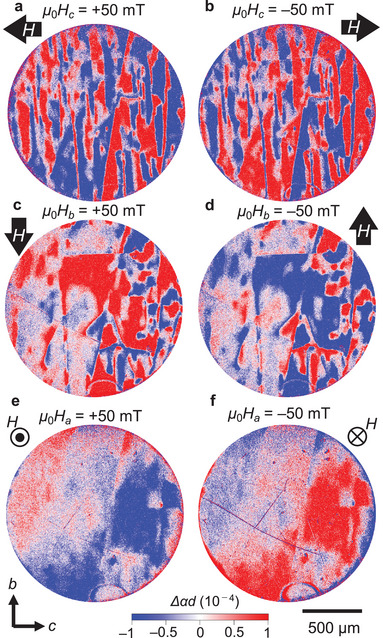
E_T_ domain inversion by applying a magnetic field. a–f) E_T_ domains in the (100) plane obtained at 4 K in the absence of a magnetic field after cooling the sample across *T*_N_ in a magnetic field of ±50 mT along the *c*‐axis, *b*‐axis, and *a‐*axis; *c*‐polarized light with a wavelength of 590 nm was used for imaging, and the applied voltage was 200 V. The domains obtained by applying positive and negative magnetic fields show the same pattern, but the contrast is inverted ((a) vs (b), (c) vs (d), and (e) vs (f)).

Because the magnetic point group *mmm* of Co_2_SiO_4_ forbids spontaneous magnetization, the E_T_ domains were expected to not respond to a magnetic field **H**. However, the domains exhibited an anomalous response to **H**. **Figure** **6** shows the domain maps on the (100) plane obtained at 4 K in the absence of **H** after cooling the samples across *T*_N_ while applying **H** of ± 50 mT along the *c* (Figure 6a,b), *b* (Figure 6c,d) and *a* (Figure 6e,f) axes. The domain strongly depends on the direction of the cooling **H**. However, a single‐domain structure has never been obtained in any **H** direction. The most surprising feature is that the domain contrasts are completely inverted by reversing the direction of cooling **H** while maintaining the positions of the domain boundaries (compare Figures 6a with 6b, Figure 6c with 6d, and Figure 6e with 6f). We also checked how the domains respond when **H** is applied below *T*_N_ (Figure S4, Supporting Information). Figure S4 in Note S7 (Supporting information) shows the result for the (100) plane sample at 49 K (just below *T*_N_) in **H**||*c*. The application of **H** changes the domain structure, and, again, the domain contrast was inverted depending on the sign of **H**.

This domain inversion in response to the flipping of the sign of **H** provides insights into the mechanism of the unexpected response of the E_T_ domains. Similar domain inversion has been reported in some multiferroics (e.g., Mn_2_GeO_4_ and Dy_0.7_Tb_0.3_FeO_3_) in which three order parameters (AFM order parameter *L*, magnetization *M*, and electric polarization *P*) are coupled via a trilinear coupling, and the Landau free energy is described as *F* ∝ −*LMP*.^[^ ^49^, ^50^, ^51^ ^]^ When one of the order parameters, e.g., *L*, is fixed, the other two (*M* and *P*) are coupled to minimize the free energy, and the domain switching of *M* accompanies that of *P*. In this case, the domain pattern of *P* is clamped by that of *L*, but the sign of *P* in each domain is inverted in response to a sign reversal of *M*.

In the case of the T‐odd antiferromagnet Co_2_SiO_4_, *L* is the only well‐defined order parameter, which is equivalent to the order parameter of the E_T_ domain. However, considering the observed response to **H**, magnetization can be another order parameter. In fact, our magnetization measurements revealed tiny spontaneous magnetization below *T*_N_ (Figure S5 in Note S8, Supporting Information). Although the observed spontaneous magnetization is very small (on the order of 10^−4^ μ_B_/f.u.), its emergence contradicts the magnetic point group of Co_2_SiO_4_ (Note S3, Supporting Information).

**Figure 7:**
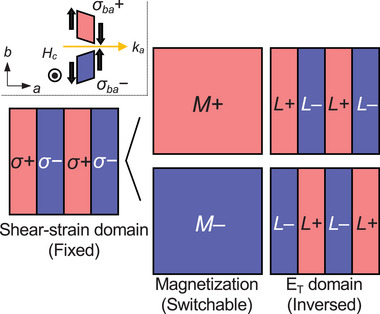
E_T_ domain inversion model with a trilinear coupling. Red and blue represent the positive and negative domain states for each order parameter (shear strain: *σ*, magnetization: *M*, E_T_ domain: *L*), respectively. The shear‐strain domain remains rigid and invariant under a magnetic field (left panel). Thus, when a magnetic field is applied and magnetization is uniform (M+ or M−), the E_T_ domain pattern mirrors the shear‐strain distribution. The sign of E_T_ domain is inverted for M+ and M−, to satisfy the trilinear coupling of *F *= −*σML*. The inset figure shows the situation corresponding to Figure 7a, where the (100) plane domain is obtained with a magnetic field applied along the *c*‐axis. In this case, the distribution of *σ_ba_* determines the E_T_ domain structure.

Here we consider that the origin of magnetization is ascribed to a linear piezomagnetic effect, a linear coupling between magnetization and strain *σ*, which is permitted in T‐odd antiferromagnets characterized by the magnetic toroidal monopole or magnetic octupole.^[^ ^30^ ^]^ In fact, the piezomagnetic effect is observed in several T‐odd antiferromagnets, including the spin reorientation phase of DyFeO_3_^[^ ^52^ ^]^ whose magnetic point group is *mmm* and the same as Co_2_SiO_4_. The linear piezomagnetic effect is defined as *M_i_* = *Λ*_*ijk*_ *σ*_*jk*_^[^ ^53^, ^54^, ^55^ ^]^ where *Λ*_*ijk*_ is a piezomagnetic tensor. In the magnetic point group *mmm*, *Λ*_*abc*_, *Λ*_*bca*_, and *Λ*_*cab*_ are finite, and their sign depends on the E_T_ domain state^[^ ^43^ ^]^ (Note S3, Supporting Information). Thus, if there is an internal shear strain in Co_2_SiO_4_, a trilinear coupling described as *F*  = −*M_i_* σ_*jk*_ *L* is expected. This strain may be induced by cutting or polishing the sample and/or intrinsic distortion of the crystal lattice. When shear‐stain domains are spatially distributed and remain unchanged during the application of **H**, the observed inversion of E_T_ domains caused by the application of **H** is well explained (**Figure** **7**). Because the application of **H** transforms the *M* domain into a single domain state, the shear‐strain and E_T_ domains are clamped, and a reversal of *M* leads to the inversion of E_T_. The direction of **H** determines which shear‐strain distributions respond (*σ_ab_* for *H_c_*, *σ_ca_* for *H_b_*, and *σ_bc_* for *H_a_*). Thus, as observed in our sample, the E_T_ domain structure depends on the direction of **H** (compare Figure 6a,c,e). The presence of the strain domains in our samples was not confirmed experimentally, which will be for future work.

## 4 Conclusion

In conclusion, we investigated the *E*‐induced NDD in the T‐odd antiferromagnet Co_2_SiO_4_. This effect is unique to materials characterized by the order of the T‐odd scalar quantity, such as the magnetic toroidal monopole. In such materials, the anomalous Hall effect and the spontaneous magneto‐optical Kerr effect do not manifest, for which reason these materials have been less investigated. As proved in this study, the *E*‐induced NDD is highly effective for revealing the order of the T‐odd scalar quantity, more particularly visualizing domains. The *E*‐induced NDD occurs in an insulator to which an electric field can be applied. Until now, discussions on T‐ and PT‐odd antiferromagnets, particularly those centered on altermagnets, have mainly focused on metallic systems.^[^ ^16^, ^17^ ^]^ Our research contributes to the extension of such studies to insulators. Candidate materials are DyFeO_3_^[^ ^52^ ^]^ and Mn_2_GeO_4_.^[^ ^49^ ^]^

The domain imaging using *E*‐induced NDD revealed a unique domain inversion in Co_2_SiO_4_ by applying a magnetic field, which cannot be identified by macroscopic property measurements. This response can be explained by a trilinear coupling mediated by the piezomagnetic effect. This suggests that E_T_ domains can be controlled through the simultaneous application of shear stress and a magnetic field. Furthermore, in systems where the piezomagnetic effect is permitted, magnetic linear dichroism emerges,^[^ ^55^ ^]^ and as its inverse effect, E_T_ domains may be controlled via irradiation with linearly polarized light in a magnetic field.^[^ ^56^ ^]^

## 5 Experimental Section

### Sample Preparation

A Co_2_SiO_4_ single crystal was grown by the floating zone method.^[^ ^57^ ^]^ The obtained crystal was oriented using Laue X‐ray diffraction, and three plate‐shaped samples with the widest faces perpendicular to the *a*, *b*, and *c* axes were prepared. For the transmittance optical measurements under an electric field, the plate‐shaped samples were polished to a thickness of 50–90 µm, and indium–tin oxide was sputtered onto the widest faces of the samples to form a pair of transparent electrodes. Magnetization was measured using a commercial physical property measurement system (PPMS, Quantum Design).

### Spatial Distribution Measurements of *E*‐Induced NDD

2D maps of the *E*‐induced NDD were obtained by using an electric field‐modulation imaging technique.^[^ ^47^ ^]^ In these measurements, a square‐wave voltage was applied to a sample, and transmission microscope images were captured using an sCMOS camera (pco edge 5.5, PCO) under alternating positive (+*V*) and negative (−*V*) voltages. Then, the difference in signals under +*V* and −*V* [Δ*I = I*(*+V*)*−I*(*−V*)] divided by their average (*I*) was calculated for each pixel. Using Equations (1) and (2), Δ*I*/*I* may be expressed as

(3) $$\begin{array}{c} \frac{\Delta I}{I}≅\frac{I_{0}e^{-\alpha_{0}d}\left(1-\Delta \alpha d\right)-I_{0}e^{-\alpha_{0}d}\left(1+\Delta \alpha d\right)}{\{I_{0}e^{-\alpha_{0}d}\left(1+\Delta \alpha d\right)+I_{0}e^{-\alpha_{0}d}\left(1-\Delta \alpha d\right)\}/2}\\ =-2\Delta \alpha d=-2\beta V \end{array}$$

To obtain spatial distributions of small signals of Δ*I*/*I* while minimizing noise, large numbers (5000–15000) of Δ*I*/*I* maps were captured and averaged. A square‐wave bias voltage was applied at a frequency of 20 Hz, and images were captured at 40 fps.

The sample temperature was controlled using a liquid He flow cryostat (Microstat He, Oxford Instruments). Domain imaging under a magnetic field was performed using an electromagnet (3470, GMW associate).

### Spectral Measurements of Optical Absorption and *E*‐Induced NDD

Optical absorption spectra and *E*‐induced NDD spectra were measured using a supercontinuum laser (SC‐Pro, YSL Photonics) and an acousto‐optic wavelength tunable filter (AOTF‐PRO, YSL Photonics). The spectra were measured by varying the wavelength of the incident linearly polarized light in 1 nm increments. In the absorption spectrum measurements, the intensity of light transmitted through the sample was measured and normalized using the intensity of light transmitted through only the window. In the *E*‐induced NDD spectrum measurements, signals were detected using a lock‐in technique. A sinusoidal voltage *V*_0_sin(2*πft*) was applied, where *V*_0_ = 150 V and *f *=  999 Hz. Under such a sinusoidal voltage, the intensity of the transmitted light may be expressed as

(4) $$\begin{array}{c} I_{0}e^{-(\alpha_{0}+\beta V_{0}\sin\left(2\pi ft\right)/d)d}\\ \approx I_{0}e^{-\alpha_{0}d}\left(1-\beta V_{0}\sin\left(2\pi ft\right)\right) \end{array}$$

Then, the intensity of transmitted light oscillating at the frequency *f* was detected by using a lock‐in amplifier, and Δ*α*  ≡ *β* *V*_0_/*d* was calculated. The *E*‐induced NDD spectra were measured at both E_T_+ and E_T_– domains by focusing the laser to a point smaller than the domain size (beam size ≈50 µm). In the same manner as the spatial distribution measurements, the sample temperature was controlled using the liquid He flow cryostat.

## Conflict of Interest

The authors declare no conflict of interest.

## Supporting information



Supporting Information

## Data Availability

The data that support the findings of this study are available from the corresponding author upon reasonable request.
