# Supplementary material for: Electric Field‐Induced Nonreciprocal Directional Dichroism in a Time‐Reversal‐Odd Antiferromagnet
Source: Adv Mater. 2025 Jan 15;37(9):2414876. doi: 10.1002/adma.202414876 (PMC11881677; doi:10.1002/adma.202414876)
Supplement: Supplementary file 1 — Supporting Information [file ADMA-37-2414876-s001.pdf]

ADVANCED MATERIALS

Supporting Information

for *Adv. Mater.*, DOI 10.1002/adma.202414876

Electric Field-Induced Nonreciprocal Directional Dichroism in a Time-Reversal-Odd Antiferromagnet

Takeshi Hayashida, Koei Matsumoto and Tsuyoshi Kimura**

Supporting Information

Electric Field-Induced Nonreciprocal Directional Dichroism in a Time-Reversal-Odd Antiferromagnet

Takeshi Hayashida, Koei Matsumoto, and Tsuyoshi Kimura**

T. Hayashida, K. Matsumoto, T. Kimura
Department of Applied Physics, University of Tokyo, Bunkyo-ku, Tokyo 113-8656, Japan
E-mail: thayashida@ap.t.u-tokyo.ac.jp; tkimura@ap.t.u-tokyo.ac.jp

Note S1. Phenomenology of the electrotoroidic effect

Here, we discuss phenomenological aspects of the electrotoroidic effect based on the Landau free energy. An expansion of the free energy as a power series in an electric field **E**, a magnetic field **H**, and a source vector **S** of the toroidalization (magnetic toroidal moment) **T** [as we mentioned in the main text, components of **S** are given as $S_i = (\mathbf{E} \times \mathbf{H})_i$] is given as

$$F(\mathbf{E},\mathbf{H},\mathbf{S}) = F_0 - P_i^s E_i - M_i^s H_i - T_i^s S_i - \frac{1}{2}\chi_{ij}^e E_i E_j - \frac{1}{2}\chi_{ij}^m H_i H_j - \frac{1}{2}\tau_{ij} S_i S_j - \alpha_{ij} E_i H_j - \theta_{ij} E_i S_j - \zeta_{ij} H_i S_j - \cdots \quad \text{(S1)}$$

where P^s, M^s, and T^s are the spontaneous polarization, spontaneous magnetization, and spontaneous toroidalization, respectively[1–3]. Differentiation of Equation (S1) leads to the toroidalization

$$T_i(\mathbf{E},\mathbf{H},\mathbf{S}) = -\frac{\partial F}{\partial S_i} = T_i^S + \tau_{ij} S_j + \theta_{ij} E_j + \zeta_{ij} H_j + \cdots \quad \text{(S2)}$$

where τ_{ij} is the susceptibility of toroidalization (toroidic susceptibility), θ_{ij} is the electrotoroidic (ET) tensor discussed in this work, and ζ_{ij} is the magnetotoroidic tensor.

In the following, we focus on the ET tensor θ_{ij} which is a second rank time-reversal odd ($\mathcal{T}$-odd) polar tensor. From the symmetry discussion using Neumann's principle, 58 magnetic point groups without $\mathcal{T}$ symmetry permit the ET effect (see Table S1)[4]. Among them, 32 point groups (yellow-colored rows in Table S1), including *mmm*, allow for the diagonal ET effect, which are classified into magnetic toroidal monopole[5]. Additionally, eight point groups ($4', \bar{4}', 4'/m, 4'22', 4'mm', \bar{4}'2m', \bar{4}'m2', 4'/mmm'$) allow antisymmetric diagonal components, θ_{11} and $\theta_{22} (= -\theta_{11})$, which are characterized by magnetic toroidal quadrupole and magnetic octupole[6,7]. In Table S1, magnetic point groups in red text are pyromagnetic point groups that permit spontaneous magnetization. The table clearly shows that the off-diagonal ET effect is allowed only in the pyromagnetic point groups except for the eight point groups characterized by magnetic toroidal quadrupole and magnetic octupole.

Table S1. Electrotoroidic coefficient matrix. Magnetic point groups are classified based on the ET tensor components. Point groups in yellow-colored rows allow for the diagonal ET effect. Magnetic point groups in red text are pyromagnetic point groups which permit spontaneous magnetization.

Magnetic point group	Electrotoroidic coefficient matrix
$1, \bar{1}$	$\begin{pmatrix} \theta_{11} & \theta_{12} & \theta_{13} \\ \theta_{21} & \theta_{22} & \theta_{23} \\ \theta_{31} & \theta_{32} & \theta_{33} \end{pmatrix}$
$2, m, 2/m$	$\begin{pmatrix} \theta_{11} & 0 & \theta_{13} \\ 0 & \theta_{22} & 0 \\ \theta_{31} & 0 & \theta_{33} \end{pmatrix}$
$2', m', 2'/m'$	$\begin{pmatrix} 0 & \theta_{12} & 0 \\ \theta_{21} & 0 & \theta_{23} \\ 0 & \theta_{32} & 0 \end{pmatrix}$
$222, 2mm, mmm$	$\begin{pmatrix} \theta_{11} & 0 & 0 \\ 0 & \theta_{22} & 0 \\ 0 & 0 & \theta_{33} \end{pmatrix}$
$2'2'2, 2'm'm, m'm'2, m'm'm$	$\begin{pmatrix} 0 & \theta_{12} & 0 \\ \theta_{21} & 0 & 0 \\ 0 & 0 & 0 \end{pmatrix}$
$3, \bar{3}, 4, \bar{4}, 4/m, 6, \bar{6}, 6/m$	$\begin{pmatrix} \theta_{11} & \theta_{12} & 0 \\ -\theta_{12} & \theta_{11} & 0 \\ 0 & 0 & \theta_{33} \end{pmatrix}$
$4', \bar{4}', 4'/m$	$\begin{pmatrix} \theta_{11} & \theta_{12} & 0 \\ \theta_{12} & -\theta_{11} & 0 \\ 0 & 0 & 0 \end{pmatrix}$
$422, 4mm, \bar{4}2m, 4/mmm, 32, 3m, \bar{3}m, 622$<br>$6mm, \bar{6}m2, 6/mmm$	$\begin{pmatrix} \theta_{11} & 0 & 0 \\ 0 & \theta_{11} & 0 \\ 0 & 0 & \theta_{33} \end{pmatrix}$
$4'22', 4'mm', \bar{4}'2m', \bar{4}'m2', 4'/mmm'$<br>$[4'2'2, 4'm'm, \bar{4}'m'2, \bar{4}'2'm, 4'/mm'm]$	$\begin{pmatrix} \theta_{11} & 0 & 0 \\ 0 & -\theta_{11} & 0 \\ 0 & 0 & 0 \end{pmatrix} \left[\begin{pmatrix} 0 & \theta_{12} & 0 \\ \theta_{12} & 0 & 0 \\ 0 & 0 & 0 \end{pmatrix} \right]$
$42'2', 4m'm', \bar{4}2'm', 4/mm'm', 32', 3m', \bar{3}m'$<br>$62'2', 6m'm', \bar{6}m'2', 6/mm'm'$	$\begin{pmatrix} 0 & \theta_{12} & 0 \\ -\theta_{12} & 0 & 0 \\ 0 & 0 & 0 \end{pmatrix}$
$23, m\bar{3}, 432, \bar{4}3m, m\bar{3}m$	$\begin{pmatrix} \theta_{11} & 0 & 0 \\ 0 & \theta_{11} & 0 \\ 0 & 0 & \theta_{11} \end{pmatrix}$
Other magnetic point groups	$\begin{pmatrix} 0 & 0 & 0 \\ 0 & 0 & 0 \\ 0 & 0 & 0 \end{pmatrix}$

We also mention to the relationship between the diagonal ET effect and the piezomagnetic effect. As mentioned above, **S** is given as $S_i = (\mathbf{E} \times \mathbf{H})_i$ and the ET coupling term $\theta_{ij}E_iS_j$ in Equation (S1) is a special cases of the bilinear magnetoelectric effect with the terms of $\gamma_{ijk}E_iE_jH_k$, where γ_{ijk} is a third rank $\mathcal{T}$-odd axial tensor and called magnetobielectric tensor. On the other hand, the piezomagnetic effect is described as $\sigma_{ij} = \Lambda_{ijk}H_k$, and Λ_{ijk} is also a third rank $\mathcal{T}$-odd axial tensor. Therefore, when the ET effect is allowed, the piezomagnetic effect is always allowed (see also Note S3). In the present case of Co_2SiO_4, there are three independent ET tensor terms of θ_{11}, θ_{22}, and θ_{33}, leading to the independent terms of the magnetobielectric tensor of γ_{123}, γ_{231}, and γ_{312} and the piezomagnetic tensor of Λ_{123}, Λ_{231}, and Λ_{312}.

Note S2. Nonreciprocal directional dichroism induced by the ET effect

Here we show how the electric field-induced nonreciprocal directional dichroism (*E*-induced NDD) is derived under the emergence of the diagonal ET effect, based on the discussions on the optical magnetoelectric effect[8–11]. In the following discussion, we assume that light propagates along the *c* axis (the third axis) and an electric field is also applied along the *c* axis (E_3). In this setting, the oscillating polarization $\mathbf{P}^{\omega}$ (magnetization $\mathbf{M}^{\omega}$) induced by oscillating magnetic field $\mathbf{H}^{\omega}$ (oscillating electric field $\mathbf{E}^{\omega}$) of light is described as

$$\begin{aligned} P_i^{\omega} &= \epsilon_0[\epsilon_{ii}(\omega) - 1]E_i(\omega) + \sqrt{\epsilon_0/\mu_0}\,\chi_{ij}^{\mathrm{em}}(\omega)H_j(\omega) \\ &= \epsilon_0[\epsilon_{ii}(\omega) - 1]E_i(\omega) + \sqrt{\epsilon_0/\mu_0}\,\gamma_{ij3}^{\mathrm{eme}}(\omega)H_j(\omega)E_3 \end{aligned} \tag{S3}$$

$$\begin{aligned} M_j^{\omega} &= [\mu_{jj}(\omega) - 1]H_j(\omega) + \sqrt{\epsilon_0/\mu_0}\,\chi_{ji}^{\mathrm{me}}(\omega)E_i(\omega) \\ &= [\mu_{jj}(\omega) - 1]H_j(\omega) + \sqrt{\epsilon_0/\mu_0}\,\gamma_{ji3}^{\mathrm{mee}}(\omega)E_i(\omega)E_3 \end{aligned} \tag{S4}$$

where $i = 1$ and $j = 2$ or $i = 1$ and $j = 2$, depending on the polarization of light, ϵ_{ij} and μ_{ij} are the dielectric permittivity and the magnetic permittivity tensor, respectively, and ϵ_0 and μ_0 are the dielectric permittivity and the magnetic permittivity of vacuum, respectively. In the magnetic point group *mmm* discussed in this work, there are three independent diagonal components of ϵ_{ij} (μ_{ij}); ϵ_{11}, ϵ_{22}, and ϵ_{33} (μ_{11}, μ_{22}, and μ_{33}). Thus, there are also three independent diagonal components in the refractive index, N_{11}, N_{22}, and N_{33}. $\gamma_{ij3}^{\mathrm{mee}}(\omega)$ and $\gamma_{ji3}^{\mathrm{eme}}(\omega)$ are the optical bilinear magnetoelectric tensors and their symmetry is the same with the static one discussed above. Note that $\gamma_{123}^{\mathrm{mee}}(\omega) \neq \gamma_{213}^{\mathrm{mee}}(\omega)$ in the magnetic point group *mmm*. Then by solving the Maxwell equations with incorporating the optical bilinear magnetoelectric introduced above, nonreciprocal component ($\Delta\alpha_{\mathrm{NDD}}$) of the absorption is described as[10,11]

$$\Delta\alpha_{\mathrm{NDD}} = \frac{2\omega}{c}\mathrm{Im}[N_{+}(\omega) - N_{-}(\omega)] \approx \frac{2\omega}{c}\mathrm{Im}\left[\chi_{ji}^{\mathrm{me}}(\omega) + \chi_{ij}^{\mathrm{em}}(\omega)\right]$$
$$= \frac{2\omega}{c}\mathrm{Im}\left[\gamma_{ji3}^{\mathrm{mee}}(\omega) + \gamma_{ij3}^{\mathrm{eme}}(\omega)\right]E_3, \tag{S5}$$

where c is speed of light and $N_{\pm}(\omega)$ is the complex refractive index for light propagating along the $\pm c$ direction. Then the simple expressions of the E-induced NDD as the changes in α under **E** is obtained as shown in Eq. (1) in the main text.

Note S3. Electrotoroidic, pyromagnetic, and piezomagnetic coefficients for magnetic point group *mmm*

Based on Neumann's principle, we derive the tensor elements of the electrotoroidic, pyromagnetic, and piezomagnetic tensors for the magnetic point group *mmm* which has three mirror symmetry elements, $m_{\perp a}, m_{\perp b}$, and $m_{\perp c}$. In matrix forms, these symmetry operations are given by

$$\begin{pmatrix} a_{11} & a_{12} & a_{13} \\ a_{21} & a_{22} & a_{23} \\ a_{31} & a_{32} & a_{33} \end{pmatrix} = \begin{pmatrix} -1 & 0 & 0 \\ 0 & 1 & 0 \\ 0 & 0 & 1 \end{pmatrix} \text{ for } m_{\perp a}, \begin{pmatrix} 1 & 0 & 0 \\ 0 & -1 & 0 \\ 0 & 0 & 1 \end{pmatrix} \text{ for } m_{\perp b}, \begin{pmatrix} -1 & 0 & 0 \\ 0 & 1 & 0 \\ 0 & 0 & 1 \end{pmatrix} \text{ for } m_{\perp c}.$$

The electrotoroidic effect is expressed as

$$T_i = \theta_{ij}E_j$$

where T_i is magnetic toroidal moment (first rank $\mathcal{T}$-odd polar tensor) and E_j is an electric field (first rank $\mathcal{T}$-even polar tensor). The proportional coefficient θ_{ij} is electrotoroidic coefficient that is a second rank $\mathcal{T}$-odd polar tensor. Using Neumann's principle, the second rank $\mathcal{T}$-odd polar tensor, electrotoroidic coefficient θ_{ij}, transforms as follows.

$$\theta'_{il} = \pm a_{ij}a_{lk}\theta_{jk}.$$

Since θ_{ij} is a $\mathcal{T}$-odd tensor, its sign changes when the time-reversal operation is included in the transformation. This is denoted by the sign $\pm$ (with + for $\mathcal{T}$-even operations and – for $\mathcal{T}$-odd operations). Here we apply Neumann's principle to the electrotoroidic effect for the point group *mmm*. For $m_{\perp a}$,

$$(\theta') = \begin{pmatrix} \theta_{11}' & \theta_{12}' & \theta_{13}' \\ \theta_{21}' & \theta_{22}' & \theta_{23}' \\ \theta_{31}' & \theta_{32}' & \theta_{33}' \end{pmatrix} = (+1)\begin{pmatrix} -1 & 0 & 0 \\ 0 & 1 & 0 \\ 0 & 0 & 1 \end{pmatrix}\begin{pmatrix} \theta_{11} & \theta_{12} & \theta_{13} \\ \theta_{21} & \theta_{22} & \theta_{23} \\ \theta_{31} & \theta_{32} & \theta_{33} \end{pmatrix}\begin{pmatrix} -1 & 0 & 0 \\ 0 & 1 & 0 \\ 0 & 0 & 1 \end{pmatrix}$$
$$= \begin{pmatrix} \theta_{11} & -\theta_{12} & -\theta_{13} \\ -\theta_{21} & \theta_{22} & \theta_{23} \\ -\theta_{31} & \theta_{32} & \theta_{33} \end{pmatrix}.$$

From Neumann' principle, $(\theta') = (\theta)$, and therefore $\theta_{12} = \theta_{13} = \theta_{21} = \theta_{31} = 0$. In the same manner, $\theta_{12} = \theta_{21} = \theta_{23} = \theta_{32} = 0$ for $m_{\perp b}$ and $\theta_{13} = \theta_{23} = \theta_{31} = \theta_{32} = 0$ for $m_{\perp c}$. Therefore, the electrotoroidic matrix is obtained as

$$(\theta) = \begin{pmatrix} \theta_{11} & 0 & 0 \\ 0 & \theta_{22} & 0 \\ 0 & 0 & \theta_{33} \end{pmatrix}.$$

Thus, only the diagonal components of electrotoroidic tensor are allowed in the magnetic point group *mmm*.

The pyromagnetic effect is expressed as

$$M_i = q_i \Delta T$$

where M_i is the variation of spontaneous magnetization (first rank $\mathcal{T}$-odd axial tensor) responding to temperature change ΔT (zero rank $\mathcal{T}$-even tensor). The proportional coefficient q_i is pyromagnetic coefficient that is a first rank $\mathcal{T}$-odd axial tensor. In magnetic systems with spontaneous magnetization, q_i becomes finite as pyroelectric coefficient in ferroelectrics. Using Neumann's principle, the first rank $\mathcal{T}$-odd axial tensor, pyromagnetic coefficient q_i, transforms as follows.

$$q_i' = \pm |a| a_{ij} q_j$$

where the sign $\pm$ denotes the absence or presence of time-reversal operation in the transformation. $|a|$ is the determinant of the transformation matrix. We apply Neumann's principle to the pyromagnetic effect of the magnetic point group *mmm*. For $m_{\perp a}$,

$$(q') = \begin{pmatrix} q_1' \\ q_2' \\ q_3' \end{pmatrix} = (+1)(-1) \begin{pmatrix} -1 & 0 & 0 \\ 0 & 1 & 0 \\ 0 & 0 & 1 \end{pmatrix} \begin{pmatrix} q_1 \\ q_2 \\ q_3 \end{pmatrix} = \begin{pmatrix} q_1 \\ -q_2 \\ -q_3 \end{pmatrix}.$$

From Neumann' principle, $(q') = (q)$, and therefore, $q_2 = q_3 = 0$. In the same manner, $q_1 = q_3 = 0$ for $m_{\perp b}$ and $q_1 = q_2 = 0$ for $m_{\perp c}$. Therefore, the pyromagnetic matrix is obtained as

$$\begin{pmatrix} q_1 \\ q_2 \\ q_3 \end{pmatrix} = \begin{pmatrix} 0 \\ 0 \\ 0 \end{pmatrix}.$$

Thus, spontaneous magnetization is not permitted in any direction for the magnetic point group *mmm*.

The piezomagnetic effect is expressed as

$$M_i = \Lambda_{ijk} \sigma_{jk}$$

where σ_{jk} is strain (second rank $\mathcal{T}$-even polar tensor) and the proportional coefficient Λ_{ijk} is piezomagnetic coefficient that is a third rank $\mathcal{T}$-odd axial tensor. Using Neumann's principle, the third rank $\mathcal{T}$-odd axial tensor, pyromagnetic coefficient q_i, transforms as follows.

$$\Lambda'_{imn} = \pm|a|a_{ij}a_{mk}a_{nl}\Lambda_{jkl}.$$

In a similar manner to the above derivations, the piezomagnetic matrix is obtained as

$$(\Lambda) = \begin{pmatrix} 0 & 0 & 0 & \Lambda_{14} & 0 & 0 \\ 0 & 0 & 0 & 0 & \Lambda_{25} & 0 \\ 0 & 0 & 0 & 0 & 0 & \Lambda_{36} \end{pmatrix}$$

where the subscripts 14, 25, and 36 corresponds to 123, 231, and 312, respectively. Thus, Λ_{abc}, Λ_{bca}, and Λ_{cab} can be finite in the magnetic point group *mmm*.

There are 66 magnetic point groups which permit the piezomagnetic effect. All the 58 electrotoroidic point groups listed in Table S1 belong to the piezomagnetic point groups. Thus, materials showing the ET effect allows the piezomagnetic effect, too.

Note S4. Calculation of *E*-induced NDD from the domain images

The white boxes in Figures S1a, S1b, and S1c show the areas where the average of Δ*ad* was obtained to calculate the voltage dependence of *E*-induced NDD in Figures 4d, 4h, and 4l in the main text, respectively.

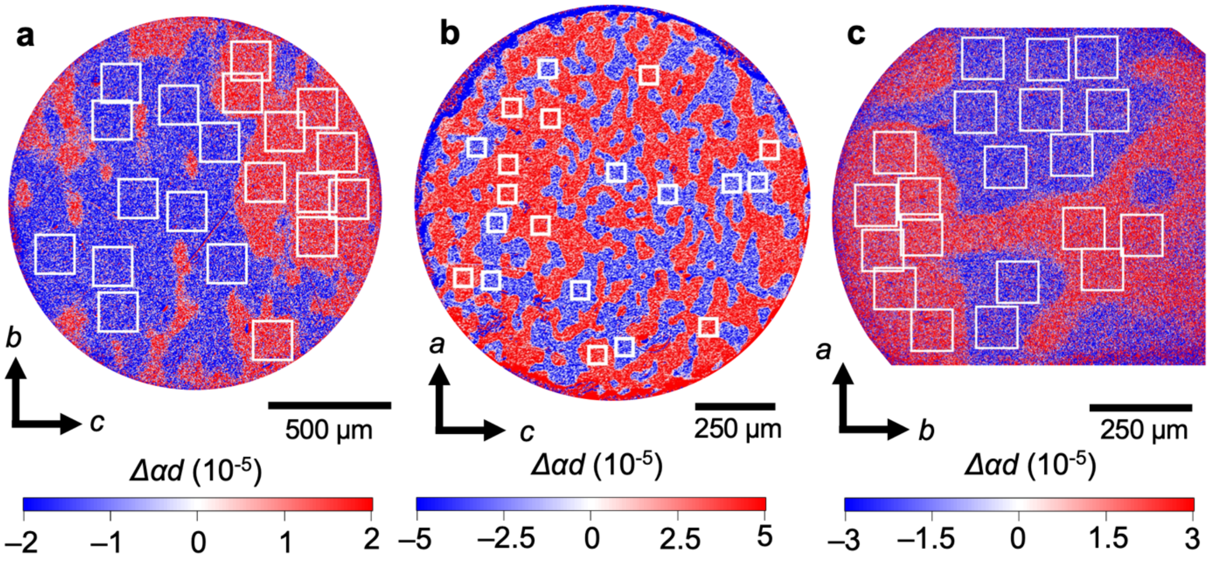

Figure S1. Sampling positions for calculating the voltage dependence of *E*-induced NDD.

Note S5. Temperature dependence of *E*-induced NDD

The temperature dependence of $\Delta\alpha$ at 150 V was obtained by focusing a laser beam on a single domain region and using a lock-in technique, with the sample temperature increasing at a rate of 1 K/min (Figure S2). Considering the results of the spectral measurements (see Figure S3), the wavelengths that maximized $\Delta\alpha$ were selected (588 nm for Figures S2a,f, 587 nm for Figures S2b,e, 589 nm for Figures S2c,d). Except for Figure S2a, the signals of *E*-induced NDD disappear around 50 K, corresponding to T_N. In Figure S2a, the signal disappears at a lower temperature (≈ 47 K), which is attributed to heating caused by irradiation with the focused laser.

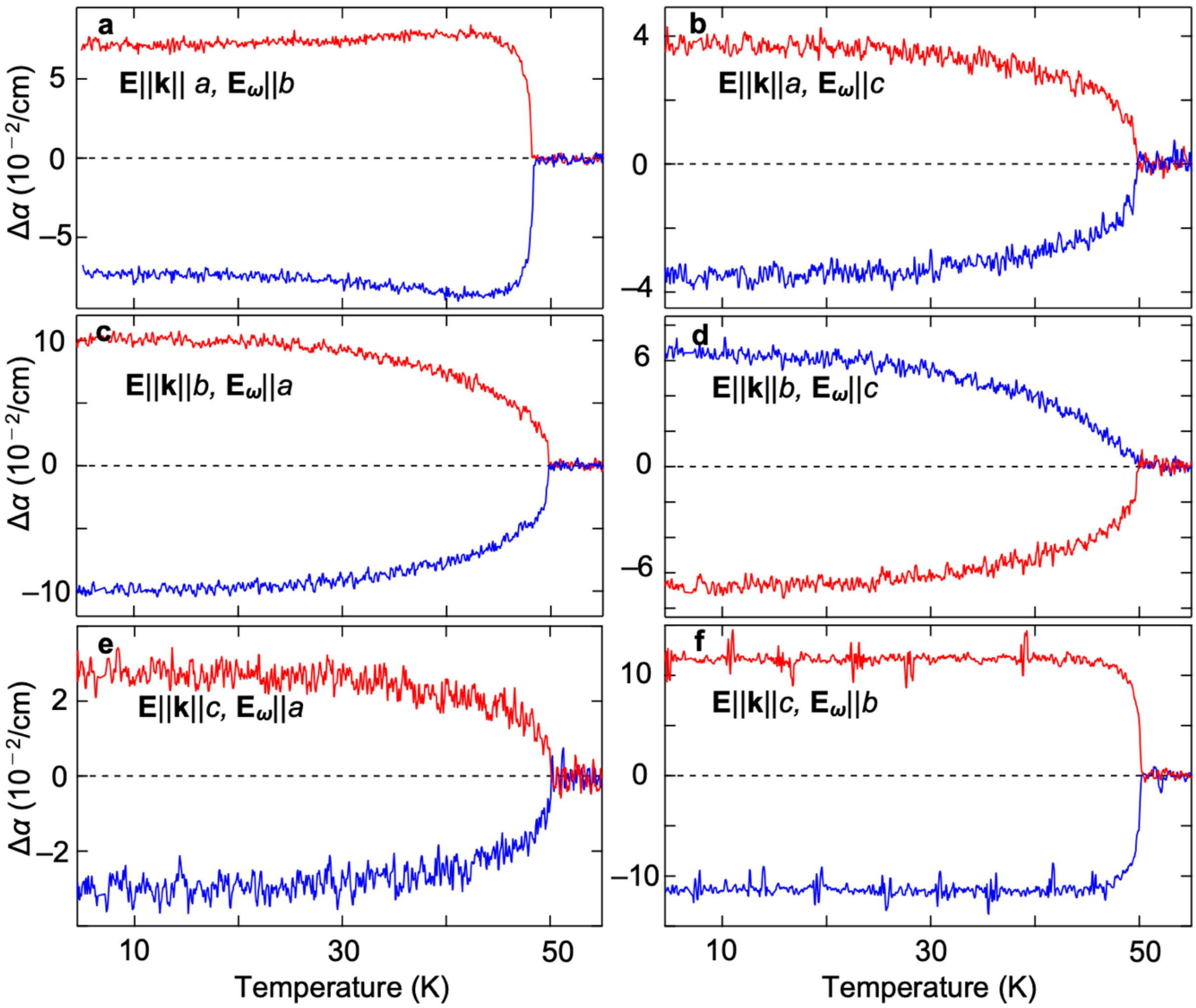

Figure S2. Temperature dependence of E-induced NDD. The temperature dependence of $\Delta\alpha$ at 150 V was obtained by focusing a laser beam on a single domain region and using a lock-in technique, with the sample temperature increasing at a rate of 1 K/min. The wavelengths that maximized $\Delta\alpha$ were selected [588 nm for (a),(f), 587 nm for (b),(e), 589 nm for (c),(d)]. Panels (a) [(b)] show the temperature dependence of α and $\Delta\alpha$ for b[c]-polarized light ($\mathbf{E}_\omega||b[c]$) propagating along the a axis ($\mathbf{k}||a$). In the same manner, panels (c)[(d)] show the temperature dependence for $\mathbf{k}||b$ and $\mathbf{E}_\omega||a[c]$, while panels (e)[(f)] show those for $\mathbf{k}||c$ and $\mathbf{E}_\omega||a[b]$. The red and blue correspond to the data obtained with either E_T+ or E_T– domains. The results in two different $\mathbf{E}_\omega$ for the same $\mathbf{k}$ obtained at the same domain state are represented by the same color.

Note S6. Energy dependence of *E*-induced NDD

In the followings, we discuss the details of *E*-induced NDD spectrum. Here, the measurements in the two polarization settings of each **k** direction were performed for the same domain state, and the red and blue dots denote the data obtained in either E_T+ or E_T– domains. Figures S3a and S3d (S3g and S3j) show the spectra of α and $\Delta\alpha$ for b(c)-polarized light [$\mathbf{E}_\omega||b(c)$] propagating along the a axis ($\mathbf{k}||a$), respectively. In the same manner, Figures S3b and S3e (S3h and S3k) show the spectra for $\mathbf{k}||c$ and $\mathbf{E}_\omega||b(a)$, while Figures S3c and S3f (S3i and S3l) show those for $\mathbf{k}||b$ and $\mathbf{E}_\omega||a(c)$. The *E*-induced NDD spectra taken at the opposite domains show a complete sign reversal (compare red and blue lines in Figures S3d-f and Figures S3j-l). In Co_2SiO_4, the absorption in the visible light region is mainly due to the Co^{2+} *d*-*d* transition[12]. Among the two Co sites, the Co1 site in a centrosymmetric field does not significantly contribute to the absorption[12]. Thus, we focus on the Co2 site in the following discussion. The absorption peaks observed around 2.0–2.6 eV are ascribed to the *d*-*d* transition from the ground state T_{1g} to the excited T_{1g} state. Here, T_{1g} refers to the state split by the cubic crystal field. Because of the effect of the distorted crystal field, it is further split into roughly three states. Around the same energy, the *E*-induced NDD shows complicated peak structures. In the following discussion, we do not delve into the details of all the peaks but consider a model to explain the origin of *E*-induced NDD in each setting from the overall features of the spectra.

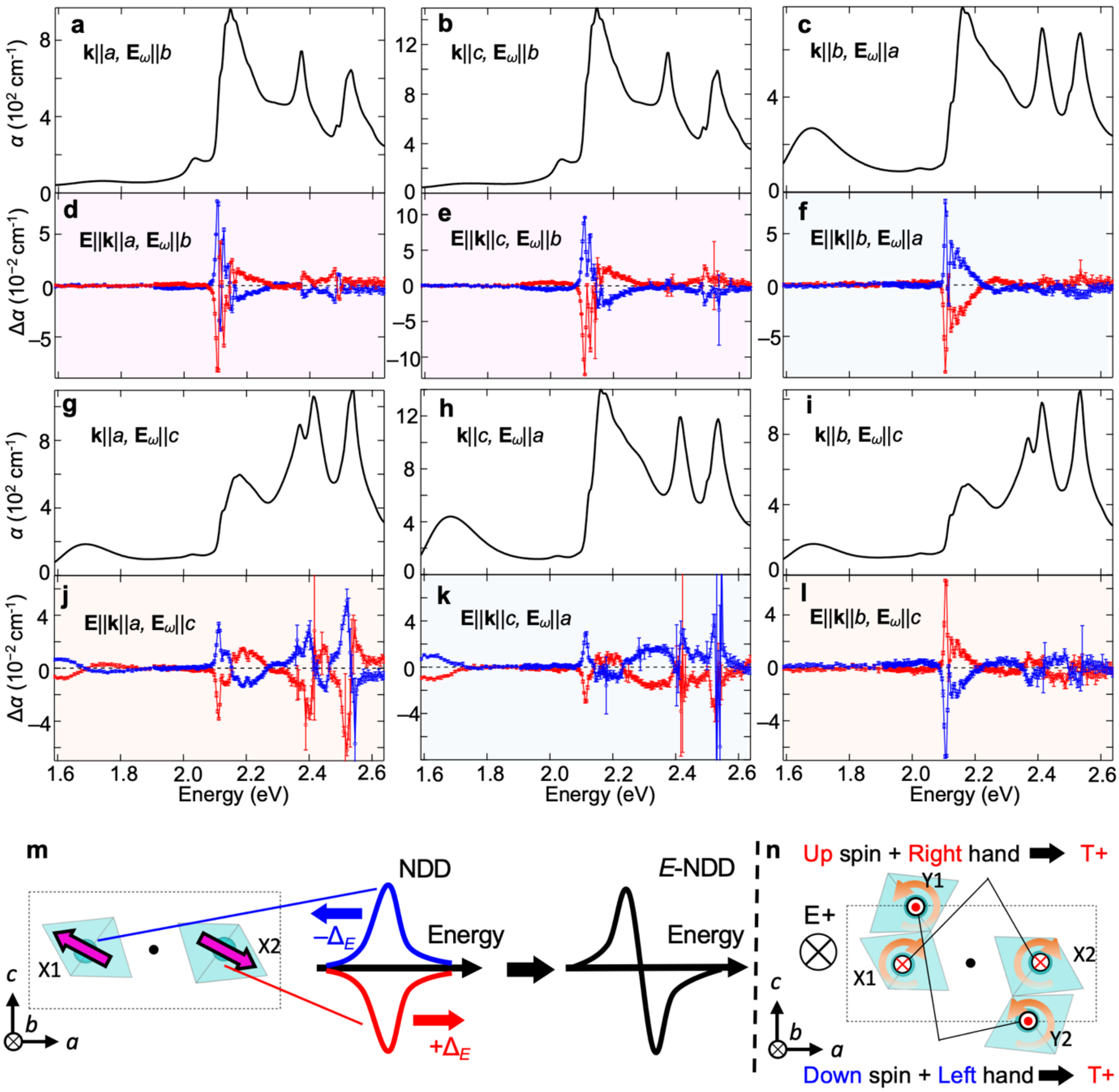

Figure S3. Spectra of *E*-induced NDD a)-l) Energy dependence of absorption coefficient α [(a)-(c) and (g)-(i)] and *E*-induced NDD $\Delta\alpha$ under 150 V [(d)-(f) and (j)-(l)] at 4 K. Here, for comparison with α, *E*-induced NDD is shown as $\Delta\alpha$, not $\Delta\alpha d$. Panels (a) and (d) [(g) and (j)] show the spectra of α and $\Delta\alpha$ for *b*[*c*]-polarized light ($\mathbf{E}_\omega||b[c]$) propagating along the *a* axis ($\mathbf{k}||a$), respectively. In the same manner, panels (b) and (e) [(h) and (k)] show the spectra for $\mathbf{k}||c$ and $\mathbf{E}_\omega||b[a]$, while panels (c) and (f) [(i) and (l)] show those for $\mathbf{k}||b$ and $\mathbf{E}_\omega||a[c]$. In the $\Delta\alpha$, spectra, the electric field **E** is applied parallel to **k**. The red and blue dots in the *E*-induced NDD spectra correspond to the data obtained for either E_T+ or E_T– domains. The results in two different $\mathbf{E}_\omega$ for the same **k** obtained at the same domain state are represented by the same color. The $\Delta\alpha$ spectra for the same direction of $\mathbf{E}_\omega$ are highlighted in the same color. Error bars show standard deviations. m) Model for the *E*-induced NDD in the case of $\mathbf{E}||\mathbf{k}||a$ or *c*. n) Model for the *E*-induced toroidal moment in the case of $\mathbf{E}||\mathbf{k}||b$.

An important feature is that the spectra at **E**||**k**||a and **E**$_\omega$||b (Figure S3d) and those at **E**||**k**||c and **E**$_\omega$||b (Figure S3e) are quite similar, whereas those at **E**||**k**||a and **E**$_\omega$||c (Figure S3j) significantly differ from those at **E**||**k**||b and **E**$_\omega$||c (Figure S3l), and those at **E**||**k**||c and **E**$_\omega$||a (Figure S3k) from those at **E**||**k**||b and **E**$_\omega$||a (Figure S3f). The E-induced NDD spectra at **E**||**k**||a,c have finer peak structures, where positive and negative signal swings are sharper than those at **E**||**k**||b. Because no significant differences are observed in the absorption spectra (compare Figure S3g with Figure S3i), the magnetic structure rather than structural anisotropy should determine the difference in the E-induced NDD spectra. This can be considered in a model based on the local magnetic toroidal moments as follows.

Let us first discuss the model for the E-induced NDD spectra at **E**||**k**||b. As illustrated in Figure S3n, Co^{2+} spins at the Co2 site are parallel to the b axis so that a toroidal moment parallel to the b axis seems not to be induced even if **E** is applied. At finite **E**, however, the mirror plane perpendicular to the b axis at each site is broken, resulting in a chiral state. When the system is chiral, a toroidal moment parallel to a magnetic moment is induced because of magneto-chiral coupling[13,14]. Now, the two sites (X1 and X2) with up spins have the same chirality (let us assume a right-handed structure) when **E** is applied, and the other two sites (Y1 and Y2) with down spins have left-handed chirality. The X sites have up spin and right-handed chirality, and the Y sites have down spin and left-handed chirality, i.e., the X and Y sites exhibit opposite spins and chirality (see Figure S3n). Because a local magnetic toroidal moment **t** is the product of spin and chirality, the sites show the same polarity of induced **t**. Accordingly, the appearance of E-induced NDD at **E**||**k**||b can be interpreted as the sum of **t** becoming finite under **E**. With such a finite toroidal moment, the photon energy ($\hbar\omega$) dependence of NDD around a single excitation energy $\hbar\omega_{n0}$ is described as[10,11]

$$\Delta\alpha(\omega) \propto \frac{\mathrm{Re}\left[< 0\middle|m_\beta\middle|n >< n|p_\alpha|0 >\right]}{(\omega-\omega_{n0})^2+\delta_n^2}, \qquad (S6)$$

where $< 0\middle|m_\beta\middle|n >$ and $< n|p_\alpha|0 >$ are matrix elements of magnetic (m_β) and electric (p_α) dipole operators, respectively, taken between the ground $|0 >$ and the excited states $|n >$ separated by $\hbar\omega_{n0}$ energy; α and β specify the directions of the electric and magnetic fields of light, respectively; δ_n is the inverse lifetime of the excited state $|n >$. According to this equation, $\Delta\alpha(\omega)$ has an absorptive structure with a single peak at ω_{n0}, which is roughly consistent with the spectra shown in Figures S3f and S3l.

Let us now consider E-induced NDD at **E**||**k**||a,c. As mentioned above, **t** at each Co2 site is finite along the a and c axes. Although the sum of **t** in a unit cell is canceled out, the application

of **E** along a or c induces a net toroidal moment along the same direction of **E**. However, this net toroidal moment is not a major contributor to the NDD at the resonance energy. Instead, the difference between mutually antiparallel local toroidal moments is a determining factor. Let us take the X site as an example. Owing to the presence of local toroidal moments, NDD occurs locally, but the effects cancel each other out at X1 and X2. However, when **E** is applied, the excitation energy that induces $\Delta\alpha$ changes linearly with respect to **E** ($\Delta_E \propto |\mathbf{E}|$), where the sign of Δ_E is opposite at the X1 and X2 sites (pseudo-Stark effect)[15,16]. As a result, the peak energies of NDD at the X1 and X2 sites become slightly different, so that they no longer cancel out each other, resulting in a finite NDD (see Figure S3m). The same process occurs at the Y1 and Y2 sites. This behavior, taking the pseudo-Stark effect into account, may be described as follows:

$$\Delta\alpha(\omega) = \Delta\alpha^{\mathrm{X1}}(\omega) - \Delta\alpha^{\mathrm{X2}}(\omega)$$

$$\propto \mathrm{Re}\left[< 0\middle|m_\beta\middle|n >< n|p_\alpha|0 >\right]\left\{\frac{1}{[\omega-(\omega_{n0}+\Delta_E)]^2+\delta_n^2}-\frac{1}{[\omega-(\omega_{n0}-\Delta_E)]^2+\delta_n^2}\right\}$$

$$\approx \mathrm{Re}\left[< 0\middle|m_\beta\middle|n >< n|p_\alpha|0 >\right]\frac{\omega-\omega_{n0}}{[(\omega-\omega_{n0})^2+\delta^2]^2}\Delta_E. \qquad \text{(S7)}$$

In this case, $\Delta\alpha(\omega)$ is proportional to $1/(\omega-\omega_{n0})^3$ and shows a dispersive structure with two peaks of opposite signs around the resonance frequency ω_{n0}, which is roughly consistent with the spectra shown in Figures. S3d,e,j,k. Therefore, in the resonance energy region, the existence of these antiparallel toroidal moments has a stronger effect on E-induced NDD than the finite sum of toroidal moments under **E**.

Considering the above, the difference in the shape of the E-induced NDD spectra at $\mathbf{E}||\mathbf{k}||a,c$ and $\mathbf{E}||\mathbf{k}||b$ can be roughly explained. However, this explanation does not take into account the contribution of the spins on the Co1 site and the effect of orbital hybridization due to spin-orbit coupling[8], both of which could significantly influence the dispersive peak structure. A more detailed spectral analysis and theoretical calculations would be required to incorporate these factors, making this an important direction for future work.

Note S7. E_T domain inversion by sweeping a magnetic field

In the (100) plane at 49 K (just below T_N), we performed the domain imaging with sweeping the magnetic field along the c axis as 0 T → 200 mT →−200 mT→200 mT. Figure S4 shows the summary of the results. By applying just 50 mT, the domain patterns changed, as the number of domain walls perpendicular to the c axis (dw⊥c) increased (compare Figure S4a with Figure

S4b). When the magnetic field was increased to 200 mT, this tendency became more pronounced (Figure S4c). One finds that the domain contrast at 200 mT is somewhat whitish, which suggests that tiny regions with weak signals, for example, tiny multi-E_T domains smaller than the microscope resolution (several μm), spread throughout the sample. With decreasing H, the domain structure started changing again in between 50 mT and 0 T. After flipping the direction of the magnetic field, the same tendency of the increasing dw⊥c with increasing H was observed. However, the domain contrast gets inverted with maintaining the pattern from that under $+H$ (compare Figure S4e with Figure. S4c). The H dependence of $\Delta\alpha d$ averaged in one selected area surrounded by a black box in Figure S4a shows a hysteresis loop as shown in Figure S4f. The coercive magnetic field is 10 ~ 20 mT, which does not contradict the magnetization measurement where the coercive magnetic field is 5 ~ 10 mT at 49 K (see Figure S4g). The magnitude of $|M|$ increases in proportion to $|H|$, whereas the magnitude of $|\Delta\alpha d|$ begins to decrease above $|m_0H|$ = 20 mT. This is most likely because as H is increased, regions with smaller strains respond to H, forming multidomains that are smaller than the resolution limit of the microscope (for the relationship between strain and magnetic field, see the Main text).

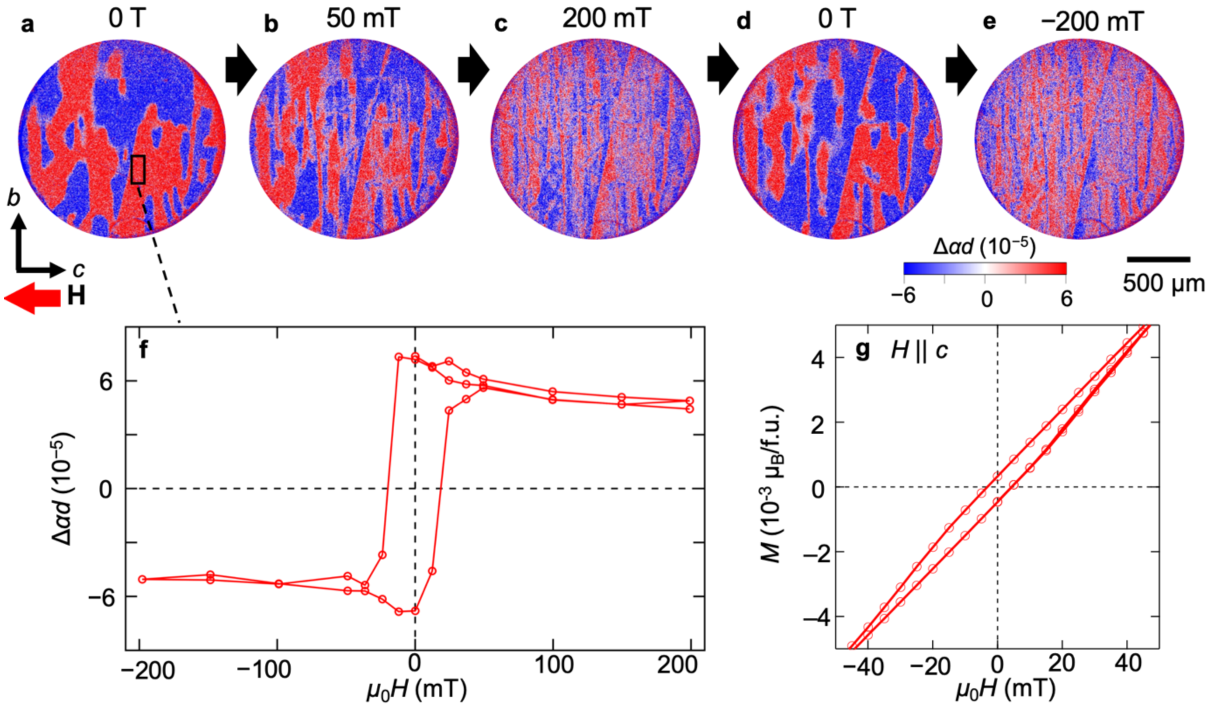

Figure S4. E_T domain inversion by sweeping a magnetic field. a)-e) E_T domains in the (100) plane obtained while sweeping the magnetic field *H* applied along the *c* axis as 0 T→200 mT→−200 mT→200 mT. The measurements were performed at 49 K (< T_N). The magnetic field was kept constant during each domain imaging. The *b*-polarized light with the wavelength of 590 nm was used for the imaging and the applied voltage was 200 V. When one compares the domains at 200 mT [(c)] and −200 mT [(e)], the pattern is the same, but the contrast is inverted. f) *H* dependence of Δad averaged in the areas surrounded by the black box in panel (a). It shows a hysteresis behavior. g) Magnetization curves at 49 K for *H* along the *c* axis.

Note S8. Detailed Magnetization Measurements

Temperature dependence of spontaneous magnetization (Figures S5a-c) and magnetization curve at 49 K (just below T_N) (Figures S5d-f) were measured along the *a*, *b,* and *c* axes.

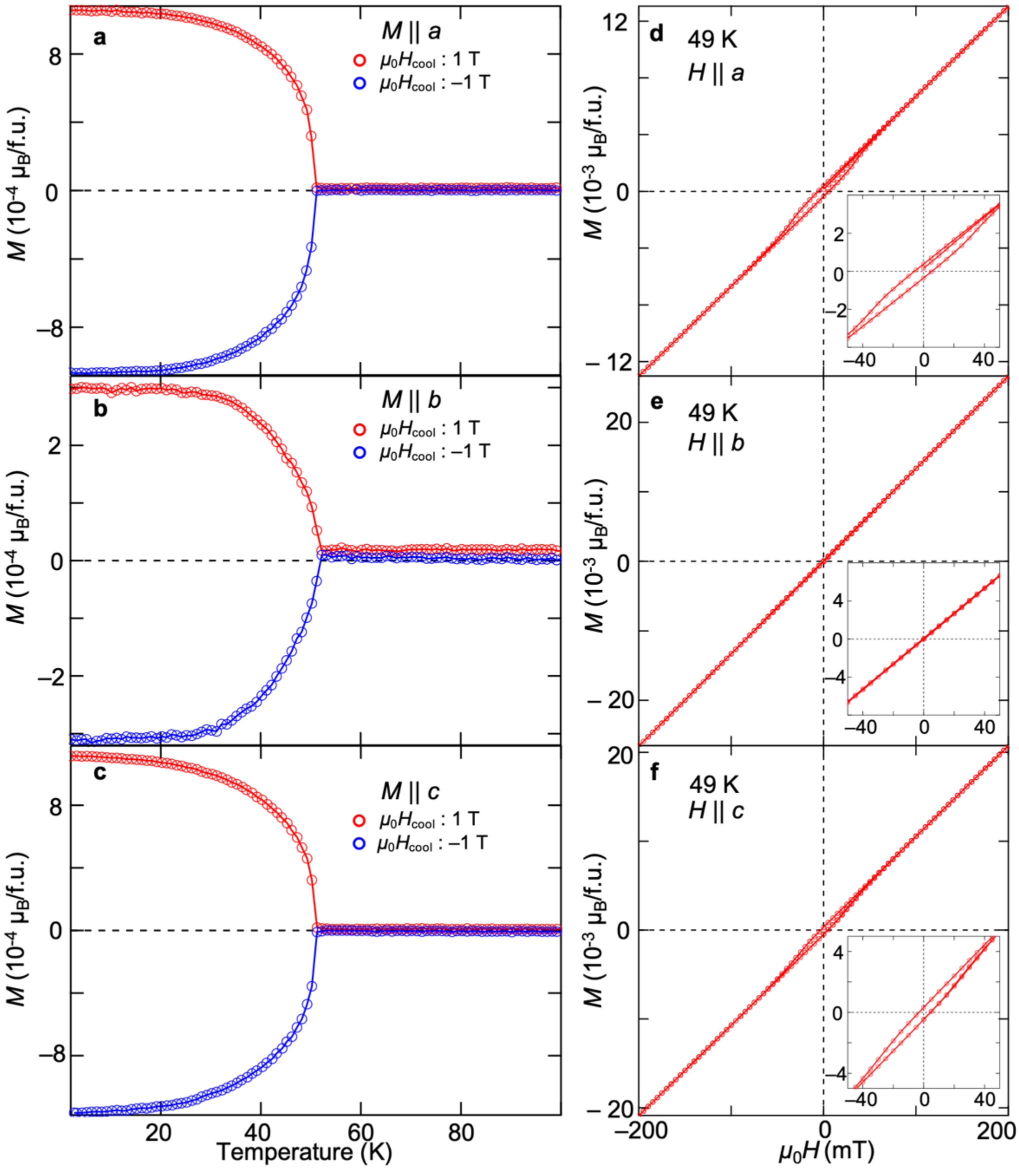

Figure S5. a)-c) Temperature dependence of spontaneous magnetization (*M*) along the *a*[(a)], *b*[(b)], and *c*[(c)] axes. The red and blue dots show the results measured after cooling the sample across T_N in a magnetic field (*H*) of 1 and –1 T, respectively. A small magnetic field up to ±0.001 T was applied during the measurements to suppress residual magnetic field. The offsets observed above T_N were attributed to residual magnetic field. d)-f) Magnetization curves at 49 K (just below T_N) for *H* along the *a*[(d)], *b*[(e)], and *c*[(f)] axes. The insets show enlarged views of the range from –50 to 50 mT. Small spontaneous magnetization and hysteresis behaviors are observed for *H* along the *a* and *c* axes, but not for *H* along the *b* axis.

Supporting References

[1] H. Schmid, *Ferroelectrics* **2001**, *252*, 41.

[2] H. Schmid, *J. Phys. Condens. Matter* **2008**, *20*, 434201.

[3] M. Fiebig, *J. Phys. D Appl. Phys.* **2005**, *38*, R123.

[4] S. V. Gallego, J. Etxebarria, L. Elcoro, E. S. Tasci, J. M. Perez-Mato, *Acta Crystallogr A* **2019**, *75*, 438.

[5] S. Hayami, H. Kusunose, *Phys. Rev. B* **2023**, *108*, L140409.

[6] M. Yatsushiro, H. Kusunose, S. Hayami, *Phys. Rev. B* **2021**, *104*, 054412.

[7] S. Hayami, M. Yatsushiro, *J. Phys. Soc. Jpn.* **2022**, *91*, 063702.

[8] T. Arima, *J. Phys. Condens. Matter* **2008**, *20*, 434211.

[9] I. Kézsmárki, N. Kida, H. Murakawa, S. Bordács, Y. Onose, Y. Tokura, *Phys. Rev. Lett.* **2011**, *106*, 057403.

[10] I. Kézsmárki, D. Szaller, S. Bordács, V. Kocsis, Y. Tokunaga, Y. Taguchi, H. Murakawa, Y. Tokura, H. Engelkamp, T. Rõõm, U. Nagel, *Nat. Commun.* **2014**, *5*, 3203.

[11] M. O. Yokosuk, H.-S. Kim, K. D. Hughey, J. Kim, A. V. Stier, K. R. O'Neal, J. Yang, S. A. Crooker, K. Haule, S.-W. Cheong, D. Vanderbilt, J. L. Musfeldt, *npj Quantum Mater.* **2020**, *5*, 20.

[12] M. N. Taran, G. R. Rossman, *Am. Mineral.* **2001**, *86*, 889.

[13] T. Sato, N. Abe, S. Kimura, Y. Tokunaga, T.-H. Arima, *Phys. Rev. Lett.* **2020**, *124*, 217402.

[14] S.-W. Cheong, S. Lim, K. Du, F.-T. Huang, *npj Quantum Mater.* **2021**, *6*, 58.

[15] W. Kaiser, S. Sugano, D. L. Wood, *Phys. Rev. Lett.* **1961**, *6*, 605.

[16] B. B. Krichevtsov, V. V. Pavlov, R. V. Pisarev, *Zh. Eksp. Teor. Fiz* **1988**, *94*, 284.
